# Long‐term functional outcomes and complications of robot‐assisted laparoscopic supratrigonal cystectomy with augmentation cystoplasty in adult patients with neurogenic lower urinary tract dysfunction and interstitial cystitis/bladder pain syndrome: A single‐centre experience

**DOI:** 10.1002/bco2.70029

**Published:** 2025-07-16

**Authors:** Thomas Batard, Benoit Mesnard, Amelie Levesque, Loïc Le Normand, Brigitte Perrouin‐Verbe, Jerome Rigaud, Marie‐Aimee Perrouin‐Verbe

**Affiliations:** ^1^ Urology Department Centre Hospitalier Universitaire de Nantes, Nantes Université Nantes France; ^2^ Physical Medicine and Rehabilitation Department Centre Hospitalier Universitaire de Nantes, Nantes Université Nantes France

**Keywords:** augmentation cystoplasty, interstitial cystitis/bladder pain syndrome, neurogenic bladder, neurogenic lower urinary tract dysfunction, robot‐assisted surgery

## Abstract

**Objectives:**

To report the long‐term functional outcomes and complications of robot‐assisted laparoscopic supratrigonal cystectomy with augmentation cystoplasty (RA‐SC‐AC) in adult patients with neurogenic lower urinary tract dysfunction (NLUTD) or Interstitial Cystitis/Bladder Pain Syndrome (IC/BPS).

**Materials and Methods:**

We retrospectively analysed the records of adult patients who underwent RA‐SC‐AC at our institution between 2012 and 2020. Patients with NLUTD had refractory neurogenic detrusor overactivity or poor bladder compliance; patients with IC/BPS presented with severe pain and/or reduced bladder capacity (<400 ml). Our centre is a national referral institution for advanced BPS/IC. We recorded early and late complications, urodynamic parameters, pain scores, continence status and quality of life (Patient Global Impression of Improvement, PGI‐I). We also report how many patients eventually required self‐catheterization.

**Results:**

Seventy‐one patients were included (41 NLUTD, 30 IC/BPS); the median follow‐up was 4.8 years ± 2.2. Overall, 36.7% experienced early (<30 days) complications, mostly minor (Clavien ≤2). Three major late complications occurred (one bladder perforation, two bowel obstructions). Among NLUTD patients, 90.2% achieved a low‐pressure reservoir, and the continence rate rose from 48.0% preoperatively to 92.7%. In IC/BPS, pain scores significantly decreased (7.8 ± 2.0 to 2.2 ± 0.4; p < 0.001) and maximum cystometric capacity increased (112 ± 39 ml to 304 ± 54 ml; p < 0.001). Four patients (13.3%) were surgical failures, persisting with severe symptoms. Eleven patients (36.7%) required de novo intermittent self‐catheterization.

Overall, 73.0% reported improved quality of life at last follow‐up.

**Conclusions:**

RA‐SC‐AC is feasible, with acceptable morbidity and long‐term functional benefits in both NLUTD and IC/BPS patients failing conservative treatments. Most patients experienced significantly improved bladder function and pain relief, as well as an enhanced quality of life.

## 1 INTRODUCTION

Supratrigonal cystectomy combined with augmentation cystoplasty (SC‐AC) involves resecting the bladder dome while preserving the trigone, ureteral orifices and bladder neck, followed by detubularized bowel augmentation. Traditionally performed via open surgery, SC‐AC is effective in addressing refractory neurogenic lower urinary tract dysfunction (NLUTD)—including detrusor overactivity or poor compliance—and, in selected cases, Interstitial Cystitis/Bladder Pain Syndrome (IC/BPS) with severely reduced capacity and/or intractable pain.^1^, ^2^, ^3^, ^4^, ^5^

However, open procedures can lead to substantial parietal morbidity and prolonged recovery.^6^, ^7^

Robot‐assisted laparoscopic approaches, sometimes combined with a mini‐laparotomy, have recently been explored to reduce wound complications and improve patient convalescence.^8^, ^9^

Nonetheless, data on robot‐assisted supratrigonal cystectomy with augmentation cystoplasty (RA‐SC‐AC) remain limited, especially regarding adult NLUTD and IC/BPS patients with end‐stage refractory symptoms.

In our single‐centre experience, we evaluated the long‐term functional outcomes, complication profile and patient satisfaction after RA‐SC‐AC in these two distinct populations.

## 2 MATERIALS AND METHODS

### 2.1 Study design and population

We retrospectively screened our database for all adult patients (≥18 years) who underwent RA‐SC‐AC between 2012 and 2020. Two main cohorts were studied: NLUTD patients (n = 41) which includes patients with refractory neurogenic detrusor overactivity (NDO) or poor bladder compliance, unresponsive to maximal conservatives measures (e.g. antimuscarinics and botulinum toxin A) and IC/BPS patients (n = 30) which includes Hunner IC/BPS to be associated with lower anatomical bladder capacity under general anesthesia (<400 ml), a higher number of Hunner lesions, and lower functional bladder capacity on voiding diaries who had failed conservative treatments (analgesics, intravesical instillations, neuromodulation).^5^

We excluded patients who underwent other major reconstructive procedures (e.g., creation of continent cutaneous diversions) simultaneously.

All cases were reviewed by a multidisciplinary team (urologists, physical medicine specialists and pain specialists).

Ethical clearance was obtained from the French Advisory Committee on Information Processing in Material Research in the Field of Health (CNIL), and all participants provided written informed consent.

### 2.2 Surgical technique and postoperative care

The surgical approach combined robot‐assisted laparoscopy (Da Vinci® system) with a small laparotomy, as previously described^9^:

Positioning and Trocars: The patient was placed in a modified lithotomy position with 25 degrees of Trendelenburg. Four trocars were placed for robotic access. An additional 12‐mm port was inserted in the left iliac fossa for the assistant.

Robot‐Assisted Supratrigonal Cystectomy and augmentation cystoplasty: Through a robotic approach, the bladder dome was opened and excised up to 2 cm from its base, preserving the trigone, bladder neck and ureteral orifices. A 5 cm mini‐laparotomy was performed and A 40 cm ileal loop was harvested through this mini‐laparotomy with restoration of intestinal continuity by end‐to‐end anastomosis using resorbable braided suture material (Vicryl 3/0). The ileal loop was detubularized and a Z‐plasty was then performed (at the surgeon's discretion) with resorbable braided suture material (Vicryl 3/0). The plasty was anastomosed to the remaining bladder through robotic approach.

A urethral catheter remained for ~3 weeks.

Postoperatively, the Foley catheter was removed upon confirming no leaks on cystography. Intermittent self‐catheterization (ISC) was initiated in case of significant post‐void residual for the BPS population and was systematically continued for the NTLUD population. Follow‐up visits were scheduled at 1, 3 and 6 months, then annually.

### 2.3 Data collection

We recorded demographic and clinical data, surgical details (operative time, length of stay) and complications. Early complications (≤30 days) and late complications (>30 days) were classified using the Clavien–Dindo system.^10^ Outcomes were analysed separately for the NLUTD and IC/BPS groups:

NLUTD: Maximum cystometric capacity (MCC), maximum detrusor pressure (P_det), bladder compliance, continence (no pad use) and medication usage.

IC/BPS: Pain (numerical rating scale 0–10), functional bladder capacity (voiding diary or urodynamics), daily frequency and analgesic requirements.

Quality of life was assessed using the Patient Global Impression of Improvement (PGI‐I). We did not formally compare baseline characteristics between questionnaire respondents and non‐respondents, which is a limitation. Patients without any visit in the last 12 months were considered lost to follow‐up.

### 2.4 Statistical analysis

Continuous variables are expressed as means ± standard deviation (SD) or medians with interquartile ranges (IQR), depending on distribution. Categorical variables are reported with counts and percentages, rounded to one decimal place. Intergroup comparisons were performed using Student's t‐test or Mann–Whitney U‐test for continuous variables, and chi‐square or Fisher's exact test for categorical variables. Significance was set at p < 0.010, with p‐values reported to three decimals (or as p < 0.001 if extremely small). Statistical analyses were carried out using IBM SPSS Statistics® (v 28.0).

## 3 RESULTS

### 3.1 Baseline characteristics

A total of 71 patients (41 NLUTD, 30 IC/BPS) were included, with a median follow‐up of 4.8 years (± 2.2). Ten patients (14.1%) were lost to follow‐up (4 NLUTD, 6 IC/BPS). Table **1** outlines the baseline demographic and clinical features, while Table 2 summarizes the preoperative management.

**TABLE 1:** Baseline characteristics of the population.

	NLUTD = 41	IC/BPS = 30
Age (years), median (IQR)	39 (± 24,5)	63,5 (± 17,5)
Gender Male number (%) Female number (%)	24 (58.5%) 17 (41.5%)	4 (13.3%) 26 (86.7%)
ASA score 1 number (%) 2 number (%) 3 number (%)	18 (43.9%) 22 (53.6%) 1 (2.5%)	14 (46.7%) 16 (53.3%) 0
BMI kg/m^2^ median, (IQR)	22,1 (± 5,1)	24 (± 3,2)
Underlying neurological disease Spinal cord injury Cervical Thoracic Lumbar Spinal Dysraphism Demyelinating Polyradiculoneuropathy Multiple Sclerosis Sacrococcygeal teratoma	27 (65.8%) 12 (44.4%) 12(44.4%) 3 (11.2%) 6 (14.6%) 1 (2,5%) 6 (14.6%) 1 (2,5%)	
Duration of the disease (years) median, (IQR)	15 (± 17,5)	6 (±6)
Continence (no pad) number, (%) Functional bladder capacity (bladder diary) (mL) median, IQR	20 (48.7%) 143 (±29)	26 (86.7%) 112 (±19)
Biological Creatinine clearance (mL/min) median (IQR) Chronic kidney failure, number (%)	101 (±18,6) 3 (7.3%)	98 (±12) 0
Pre‐operative urodynamic parameters Maximum cystometric capacity (mL) median (IQR) Detrusor overactivity, number (%) Poor bladder compliance (< 20 ml/cmH2O) number (%) Maximum detrusor pressure (cmH2O) median (IQR)) Urethral closure pressure median (IQR)	198 (±81) 38 (92.6%) 32 (78.0%) 42 (±18) 67 (±23)	132 (±39)0 2 (6.6%)28 (±12) 55 (±12)
Endoscopic parameters (for IC/BPS) Anatomical bladder capacity (ABC) under G.A. (mL) median (IQR) Macroscopic appearance of the bladder mucosa Hunner's lésion, number (%)		297 (±54) 12 (40%)

**TABLE 2:** ‐Preoperative management.

	NLUTD n (%)
Antimuscarinic drugs Botulinum Toxin A Sacral neuromodulation	41 (100.0%) 39 (95.1%) 2 (4.9%)
	**IC/BPS n (%)**
Hydrodistension (short) Hydrodistension (long) Antimuscarinic drugs Antihistamines Pentosane Polysulfate Intravesical therapy Amitriptyline Botulinum Toxin A Tibial Nerve Stimulation Sacral Neuromodulation	30 (100.0%) 9 (30.0%) 30 (100.0%) 17 (56.7%) 11 (36.7%) 6 (20.0%) 30 (100.0%) 7 (23.3%) 10 (33.3%) 6 (20.0%)

### 3.2 Peri‐operative outcomes

The overall median operative time was 231 (± 32) minutes. This time was shorter for those with IC/BPS (184 [± 49] min) than for those with NLUTD (252 [± 35] min). Of the 71 surgical procedures, no open conversion was required.

The median length of stay in the Urology Department was 8 (± 4) days for patients with IC/BPS and 7 (± 1) days for those with NLUTD. After 7 days, patients with NLUTD then transferred for rehabilitation.

The median duration of catheterization for both groups was 23 (±1) days.

### 3.3 Complications

#### 3.3.1 Early complications (≤30 days)

Overall, 26 patients (36.6%) experienced at least one early complication, most of which (Clavien–Dindo ≤2) were considered minor (e.g., febrile urinary tract infections or prolonged ileus). Three major complications (4.2%) occurred, including femoral artery thrombosis, haematuria with clot retention and urinomas requiring drainage Table 3.

**TABLE 3:** Early postoperative complications.

Complications	NLUTD	IC/BPS	Management	Grade according to Clavien‐Dindo classification
Parietal hematoma	3 (7.3%)	1 (3.3%)	None	I
Parietal abscess	1 (2.4%)	1 (3.3%)	Percutaneous drainage	II
Prolonged ileus (>7 days)	3 (7.3%)	4 (13.3%)	Nasogastric tube	
Febrile urinary tract infection = acute pyelonephritis	6 (14.6%)	2 (6.7%)	Antibiotics	
Deep vein thrombosis	1 (2,4%)	0	Oral anticoagulants	
Total clavien ≤ 2 (%)	14 (34.1%)	8 (26.7%)		
Acute Lower Limb ischemia (Femoral artery thrombosis)	0	1 (3.3%)	Catheter‐directed thrombolisis	IIIB
Hematuria with blood clots	0	1 (3.3%)	Endoscopic treatment under G.A.	
Urinoma	1 (2.4%)	1 (3.3%)	Surgery for drainage	
Total Clavien >2	1 (2.4%)	3 (10%)		

#### 3.3.2 Late complications

No deaths were reported during the follow‐up period.

In total, 25 late complications affected 16 patients (35.2%), with a reoperation rate of 1.5% (n = 1). Seven IC/BPS patients developed febrile urinary tract infections (UTIs) associated with significant post‐void residuals; all subsequently required intermittent self‐catheterization, which improved their symptoms. Febrile UTIs also occurred in four NLUTD patients, managed with antibiotics and increased self‐catheterization frequency.

New or worsening bowel dysfunction was noted in 11 NLUTD patients (26.8%). De novo constipation appeared in eight cases, generally relieved by transanal irrigation. Two patients experienced malabsorptive diarrhoea with faecal incontinence, successfully treated with cholestyramine. In the IC/BPS group, only one patient developed a new‐onset transit disorder (diarrhoea).

Bowel obstruction occurred in two patients, at 9 months and 2 years post‐surgery; both were managed conservatively with favourable outcomes. One perforation of the augmented bladder occurred 1 year postoperatively and was treated surgically with abdominal irrigation/drainage and direct repair of the defect. Throughout the study, no cases of lithiasis or neoplasms were observed.

### 3.4 Functional outcomes in NLUTD

Table 4 summarizes the urodynamic changes. At last follow‐up (median 4.8 years):

- **MCC** improved significantly from 198 ± 81 ml to 452 ± 87 ml (p < 0.001).
- **Maximum detrusor pressure** decreased from 46 ± 14 cmH₂O to 15 ± 3 cmH₂O (p < 0.001).
- **Continence** increased from 48.7% to 92.7%. To achieve continence, three patients underwent postoperative artificial urinary sphincter (with two revisions) including one patient with prior history of endoscopic sphincterotomy. One patient underwent continent cutaneous urinary diversion 3 years after AC because of difficulties to perform ISC through the native urethra.
- **Medication** usage (antimuscarinics, botulinum toxin A) dropped substantially (p < 0.010).
- **Quality of life:** Of the 29 respondents to the PGI‐I (70.7%), 72.4% indicated overall improvement. Five patients (17.2%) reported poorer quality of life due to bowel disturbances, and three (10.4%) felt that there had been no change.

**TABLE 4:** Functional outcomes in patients with NLUTD.

NLUTD N = 41	Pre‐operative	Post‐operative (3 months)	Post‐operative (last follow‐up)	P‐value
Maximum Cystometric Capacity (MCC) (mL) median (IQR)	198 (±81)	433 (±93)	452 (±87)	**<0,001**
Maximum Detrusor Pressure (PdetMax) (cmH20) median (IQR)	46 (±14)	16 (±4)	15 (±3)	**<0,001**
Poor Bladder Compliance (<20 ml/cmH2O), number (%)	32 (78.1%)	0	1 (2.4%)	**<0,001**
Neurogenic Detrusor Overactivity(n, %)	38 (92.7%)	0	1 (2.4%)	**<0,001**
Maximum urethral closure pressure, median (IQR)	82 (±34)	78 (±28)	77 (±11)	ns
Continence rate	20 (48.7%)	35 (85.4%)	38 (92.7%)	**<0,001**
Antimuscarinic drugs, number (%) Botulinum toxin A (number, %)	31 (75.6%) 39 (95.1%)	5 (12.1%) 0	7 (17.1%) 2 (4.9%)	**< 0,001** **< 0,001**

### 3.5 Functional outcomes in IC/BPS

Pre‐ and post‐operative data appear in Table 5. At last follow‐up:

- **Pain** (NPRS) decreased from 7.8 ± 2.0 to 2.2 ± 0.4 (p < 0.001).
- **Functional bladder capacity** increased from 112 ± 39 ml to 304 ± 54 ml (p < 0.001).
- **Daytime frequency** improved in most patients; 36.7% started de novo ISC due to incomplete emptying or recurrent infections.
- **Failures**: 4 patients (13.3%) experienced persistent severe symptoms with NPRS> 5/10, associated with bothersome voiding frequency. Analgesic treatment, tricyclic antidepressants and antimuscarinics were continued in these patients. Two patients underwent ganglion impar block and botulinum toxin injections into the bladder trigone without success. Non‐continent urinary diversion was suggested in these four patients. At the last follow‐up, two patients were considering this procedure, and two had been lost to follow‐up.
- Eleven patients (36.7%) had to **secondarily perform ISC** because of poor bladder emptying with urinary tract infections and/or incontinence, related to significant post‐void residual>150 ml.
- **Quality of life**: A total of 23 of the 30 (76.7%) patients with IC/BPS responded. Of them, 17 (73.9%) reported an improvement since surgery, 3 (13.05%) no change and 3 (13.05%) reported a loss of quality of life.

**TABLE 5:** Functional outcomes in patients with IC/BPS.

IC/BPS N = 30	Pre‐operative	Post‐operative (3 months)	Post‐operative (last follow‐up)	P‐value
Functional bladder capacity (mL) median (IQR)	112 (± 39)	230 (±88)	304 (±54)	**<0,001**
Voiding Frequency (>6/day)	30 (100.0%)	19 (63.3%)	7 (23.3%)	**<0,001**
Numerical pain rating scale (NPRS) median (IQR)	7,8 (±2)	1,7 (±0,8)	2,2 (±0,4)	**<0,001**
Continence rate	26 (86.7%)	21 (70.0%)	28 (93.3%)	NS
**Voiding mode** Spontaneous voiding Abdominal straining Intermittent self‐catheterization	28 (93.3%) 0 2 (6.7%)	0 27(90.0%) 3 (10.0%)	0 19 (63.3%) 11 (36.7%)	
Antimuscarinic drugs Or intradetrusor botulinum toxin A injections Number (%)	30 (100.0%)	0	2 (6.7%)	<0,001
Analgesic treatments	30 (100.0%)	12 (40.0%)	6 (20.0%)	**<0,001**

## 4 DISCUSSION

In this single‐centre retrospective series, RA‐SC‐AC yielded durable improvements in bladder storage (capacity, compliance), continence and pain relief for both NLUTD and IC/BPS populations who had exhausted conservative therapies. Early complications were mostly mild, with only three major events (4.2%) in the immediate postoperative period, and severe late complications were likewise infrequent (one bladder perforation, two bowel obstructions). These findings demonstrate a favourable risk profile, broadly comparable to open SC‐AC outcomes.^6^, ^11^, ^12^, ^13^, ^14^, ^15^, ^16^

### 4.1 Heterogeneous patient populations

Although patients with NLUTD and those with BPS/IC both can benefit from RA‐SC‐AC after failure of conservative therapies.

Because the goals differ between these populations, functional outcomes were analysed separately. For NLUTD patients, the primary objective was to establish a low‐pressure reservoir to protect the upper urinary tract and improve continence. In contrast, for BPS/IC patients, the main goal was pain relief and increased bladder capacity to address daytime frequency issues. Complications were analysed collectively.

#### 4.1.1 NLUTD

In our study, 93.0% of NLUTD patients achieved continence at last follow‐up Table 4, comparable to open‐series results^6^, ^11^, ^12^ Table 6. Additionally, we observed a marked reduction in maximum detrusor pressure and a sharp increase in bladder compliance. This suggests that robotic assistance preserves the fundamental efficacy of augmentation cystoplasty while potentially offering reduced risk of major parietal complications compared with a large laparotomy.^9^, ^17^, ^18^ Moreover, the significant decrease in antimuscarinic usage underscores the procedure's capacity to normalize bladder pressures without persistent pharmacological therapy.

**TABLE 6:** ‐literature review for NLUTD.

Reference	Approach (open/laparoscopy/robot‐assisted laparoscopy	Nb of patients and type of disease	Median follow‐up (months)	Early major postoperative complications	Late postoperative complications	Continence rate	Cystometric capacity (mL)
Herschorn et al 1998^13^	Open	73 Spinal dysraphism/SCI/sacral dysgenesis /multiple sclerosis/myopathy/degenerative ataxia	76	2%	Bladder stones 15% Metabolic disorders 4% Bladder perforation 1% Bladder cancer 1%	84%	650
Nomura et al 2002^11^	Open	21 SCI/spinal dysraphism	66	0	NR	95%	396
Khastgir et al 2003^15^	Open	32 SCI/spinal dysraphism	72	3%	Bladder stones 3% Metabolic disorders 0% Bladder perforation 3%	92%	589
Perrouin Verbe et al 2019^12^	Open	28 Spinal dysraphism	163	3.5%	Bladder stones 7% Metabolic disorders 0% Bladder perforation 4% Bladder cancer 4%	71%	486
Grilo et al 2020^17^	Robot assisted	10 SCI/Myelitis /Strumpell‐Lorrain disease/Neurosarcoidosis/Devic's disease	12	10%	Bladder stones 30% Urinary fistula 25%	100%	515

#### 4.1.2 IC/BPS

Similarly, patients with IC/BPS exhibited significant improvements in pain (NPRS from 7.8 to 2.2) and bladder capacity (112 ml to 304 ml), echoing results from previously published open supratrigonal cystectomy series^7^, ^16^ Table 7. Nonetheless, 37.0% required intermittent catheterization for incomplete emptying or infection. This highlights the importance of thorough preoperative counselling, as augmented bladders may not guarantee spontaneous voiding for all patients.

**TABLE 7:** Literature review for IC/BPS.

References	Approach (open/laparoscopy/robot‐assisted laparoscopy	Nb of patients	Hunner's lesion	Follow up (month)	Voiding frequency (>6 per day),pre and post operative		Numerical pain rating scale median pre and post operative		Intermittent self‐catheterization	Major early complications	Late complications
Kim et al 2014 ^7^	Open	40	40 (100%)	12	21,3 (±8)	9,5 (±7,7)	5	1,3	5%	5%	1 UTI; 1 Incisional hernia
Matteu Arrom et al 2019 ^16^	Open	29	4 (13%)	107	21 (±12)	10 (±4)	NR	NR	10%	20%	1 Bladder perforation; 2 Incisional herniae

The inclusion of 30 IC/BPS patients may seem unexpectedly high for such a major procedure. However, our institution is a recognized national referral centre for advanced or refractory BPS/IC, which explains the large number of patients evaluated for last‐resort surgical management. All had severely impaired bladder capacity (often <400 ml) and persistent pain (≥6/10 on a numerical rating scale), despite extensive conservative treatments (antimuscarinics, intravesical instillations, neuromodulation, etc.). In such cases, supratrigonal cystectomy with augmentation cystoplasty can provide substantial relief of pain and improve capacity, albeit at the cost of potential surgical morbidity. Our results suggest that RA‐SC‐AC can be safely applied to carefully selected IC/BPS patients, offering meaningful improvements in quality of life.

### 4.2 Limitations

Our study lacks a comparative control group (e.g., open or purely laparoscopic surgery), preventing definitive conclusions about the superiority or equivalence of RA‐SC‐AC. The sample size, particularly in subgroups, is modest, and the retrospective design introduces inherent selection bias. Nevertheless, this analysis provides insight into the real‐world application of a single surgical technique across two distinct pathologies. Larger, prospective cohorts or randomized trials, ideally with standardized protocols, are warranted to validate and refine patient selection criteria.

## 5 CONCLUSIONS

Robot‐assisted laparoscopic supratrigonal cystectomy with augmentation cystoplasty is a feasible and effective therapeutic option for patients with refractory NLUTD or IC/BPS. In our series, it resulted in durable improvements in bladder capacity, pain control and continence, with acceptable complication rates. Future controlled or randomized studies should focus on delineating specific advantages of the robotic approach and further refining patient selection criteria, especially in complex referral‐based populations.

## AUTHOR CONTRIBUTIONS

T. Batard: Protocol/project development, Data collection and management, Data analysis, Manuscript writing/editing; B. Mesnard: Data collection and management, Data analysis, Manuscript writing/editing; A. Levesque, L. Lenormand, B. Perrouin‐Verbe and J. Rigaud: Manuscript writing/editing; M.A. Perrouin‐Verbe: Protocol/project writing, Protocol/project development, Data analysis, Manuscript writing/editing.

## CONFLICT OF INTEREST STATEMENT

T. Batard, B. Mesnard, A. Levesque, L. Lenormand, and B. Perrouin‐Verbe declare no conflicts of interest related to this work. Prof. J. Rigaud and Prof. M.‐A. Perrouin‐Verbe serve as proctors for Intuitive Surgical.
